# Pattern and predictors of death from aluminum and zinc phosphide poisoning using multi-kernel optimized relevance vector machine

**DOI:** 10.1038/s41598-023-34489-x

**Published:** 2023-05-22

**Authors:** Sara Abdelghafar, Tamer Ahmed Farrag, Azza Zanaty, Heba Alshater, Ashraf Darwish, Aboul Ella Hassanien

**Affiliations:** 1 Computer Science School, Canadian International College (CIC), Cairo, Egypt; 2 Department of Computer Engineering, MISR Higher Institute for Engineering and Technology, Mansoura, Egypt; 3 Forensic Medicine and Clinical Toxicology Department, Faculty of Medicine, Menoufia University Hospital, Shibin El Kom, Egypt; 4 Forensic Medicine and Clinical Toxicology Department, Menoufia University Hospital, Shibin El Kom, Egypt; 5 Faculty of Science, Helwan University, Cairo, Egypt; 6 Faculty of Computers and Artificial Intelligence, Cairo University, Cairo, Egypt; 7 Scientific Research Group in Egypt (SRGE),, http://www.egyptscience.net

**Keywords:** Health care, Medical research

## Abstract

The use of metal phosphides, particularly aluminum phosphide, poses a significant threat to human safety and results in high mortality rates. This study aimed to determine mortality patterns and predictive factors for acute zinc and aluminum phosphide poisoning cases that were admitted to Menoufia University Poison and Dependence Control Center from 2017 to 2021. Statistical analysis revealed that poisoning was more common among females (59.7%), aged between 10 and 20 years, and from rural regions. Most cases were students, and most poisonings were the result of suicidal intentions (78.6%). A new hybrid model named Bayesian Optimization-Relevance Vector Machine (BO-RVM) was proposed to forecast fatal poisoning. The model achieved an overall accuracy of 97%, with high positive predictive value (PPV) and negative predictive value (NPV) values of 100% and 96%, respectively. The sensitivity was 89.3%, while the specificity was 100%. The F1 score was 94.3%, indicating a good balance between precision and recall. These results suggest that the model performs well in identifying both positive and negative cases. Additionally, the BO-RVM model has a fast and accurate processing time of 379.9595 s, making it a promising tool for various applications. The study underscores the need for public health policies to restrict the availability and use of phosphides in Egypt and adopt effective treatment methods for phosphide-poisoned patients. Clinical suspicion, positive silver nitrate test for phosphine, and analysis of cholinesterase levels are useful in diagnosing metal phosphide poisoning, which can cause various symptoms.

## Introduction

The use of pesticides in certain developing countries has resulted in increased agricultural productivity, but their misuse can lead to severe acute and chronic poisoning. This is a global health issue that can occur intentionally or unintentionally, with suicidal poisoning accounting for one-third of suicides worldwide^1,2^.

Globally, aluminum and zinc phosphide pesticides are commonly used to protect stored grains from pests and rodents. However, these pesticides can cause severe acute and chronic poisoning due to the release of phosphine gas when exposed to water or hydrochloric acid in the stomach. The toxic effects of phosphine gas include impaired tissue respiration, cardiac muscle toxicity, adrenal gland toxicity, and circulatory collapse, which can lead to death^3^. Aluminum Phosphide (AlP) has been reported also to cause methemoglobinemia, hemolysis, and inhibition of the cholinesterase enzyme^4–6^. Therfore, these pesticides are a significant cause of morbidity and mortality in developing countries where their use is widespread, as there is no antidote or specific treatment available^7^. Zinc Phosphide (ZnP) is a dark grey crystalline compound used as a rodenticide, which is inexpensive, easily accessible, and highly potent^8^. AlP is a solid fumigant that has been widely used since the 1940s. It is easily accessible and obtainable in developing countries. It is considered nearly ideal as it is toxic to all stages of insects, highly effective, does not affect seed viability, leaves little residue on food grains, and is free from harmful residues. This highly toxic chemical is inexpensive and commonly formulated as tablets, known as rice tablets, which contains 56% AlP and 44% aluminum carbonate^9,10^. The objective of this study is to identify the predictive variables of mortality at admission in cases of zinc and aluminum phosphide poisoning at Menoufia University Poison and Dependence Control Center. The study aims to identify patterns and predictor methods of mortality over five years. The ultimate goal is to improve patient outcomes by allowing for more intensive monitoring and treatment.

The Relevance Vector Machine (RVM) was proposed in 2001 by Tipping^11^. RVM is a predictive machine learning model that integrates Bayesian theory with fundamental principles of Support Vector Machines (SVM) to produce a method similar to SVM with probabilistic output. RVM and SVM are two popular machine-learning algorithms that are widely used in various applications of classification and regression^12–16^. Both RVM and SVM can handle non-linear relationships between input and output variables, but SVM has different types, including linear SVM, polynomial SVM, and radial basis function (RBF) SVM. Linear SVM is used when the data is linearly separable, while polynomial SVM and RBF SVM are used when the data is non-linearly separable. The choice of SVM type depends on the characteristics of the data and the problem being solved^17^. Both RVM and SVM belong to the category of kernel-based methods and work by mapping the input data into a high-dimensional feature space, where they can be separated more easily. RVM and SVM both require an optimizer for tuning their parameters, which determines the accuracy and generalization capability of the model. Many studies have introduced optimization techniques for both RVM and SVM, such as genetic algorithms^18^, grey wolf optimization^19,20^, firefly algorithm^21^, coyote optimization^22^, artificial bee colony^23^, and Bayesian optimization^24^.

RVM is a powerful and flexible probabilistic model that outperforms traditional SVMs in several ways. Unlike SVMs, which produce binary outputs, RVMs provide probabilistic outputs that are useful for applications where uncertainty is a factor. This makes RVMs more useful for applications where uncertainty is a factor, such as medical diagnosis or financial forecasting. RVMs also require fewer support vectors than SVMs, leading to faster training times and more efficient use of computational resources. Additionally, RVMs can automatically select relevant features from the input data, making them more efficient and less prone to overfitting. Overall, the RVM is a valuable tool for predictive modeling and has become increasingly popular in the machine-learning community in recent years^13,25^.

In this study, we propose a novel and optimized predictive model, named BO-RVM, which utilizes Bayesian optimization (BO) to select the optimal kernel function and estimate hyperparameters for the Relevance Vector Machine (RVM). Our experimental results demonstrate that the proposed BO-RVM model outperforms existing methods in kernel selection and hyperparameter estimation for RVM. Furthermore, we apply the BO-RVM model to predict fatal poisoning in a collected dataset, showing its reliability and potential usefulness in real-world applications.

This study makes several important contributions. Firstly, it provides a detailed analysis of zinc and aluminum phosphide poisoning cases at Menoufia University Poison and Dependence Control Center over five years, offering valuable retrospective insights. Secondly, the study identifies key predictive factors for mortality in acute poisoning cases, providing potential guidance for treatment and intervention. Thirdly, the study introduces an innovative optimized predictive model that enhances the performance of multi-kernel RVM using Bayesian optimization. Finally, the proposed BO-RVM model is rigorously evaluated for predicting fatal poisoning using the collected dataset, demonstrating its accuracy and potential usefulness in real-world applications, as evidenced by the confusion matrix..

The structure of this paper is organized as follows: section "Related work" discusses related work, while section "Basics and background" provides essential background information and relevant concepts. Section "Materials and methods" comprises the materials and methods section, which includes study design, data collection, study population, statistical analysis, and the proposed optimized predictive modeling. A comprehensive analysis of the statistical data and prediction results is presented in section "Results, discussion, and analysis". Finally, section "Conclusion and future work" summarizes the conclusions and recommendations.

## Related work

Machine learning has become a crucial tool in the medical field with a range of applications, including disease diagnosis, medical procedure recommendation, and patient behavior analysis. One study utilized the XGBoost model to predict the outcomes of mitochondrial membrane potential (MMP) and GluGal assays, which are essential indicators of mitochondrial health^10^. Another study proposed a locality-sensitive deep learner (LSDL), a deep neural network with an attention mechanism, and tested it on various datasets in the field of toxicity prediction^26^. The LSDL model outperformed other deep learning models in predicting toxicological effects. The potential of deep learning models in detecting adverse drug reactions (ADRs) was reviewed in^27^, highlighting its usefulness in pharmacovigilance. A new approach was introduced for toxic and narcotic medication detection using you only look once (YOLO) based object detectors in^28^, showing that rotated YOLO detectors are effective in identifying densely arranged drugs. Lastly, the authors developed an LSTM-based model to examine molecule toxicity, and the results showed promising performance in toxicity prediction^29^.

However, the prior studies most relevant to our study utilized a comparable dataset but relied on statistical analysis through logistic regression anaysis and the Statistical Package for the Social Science (SPSS) package instead of utilizing machine learning predictive models. Moreover, the dataset used in our proposed study is larger than those collected for their research, as shown in Table 1.

**Table 1:** Comparison of approaches and dataset sizes used in prior studies and this proposed study.

References	Method	The used approach and dataset
^3^	Logistic regression analysis and SPSS package	In this study, a prospective design was employed to collect data on patients who were admitted to Menoufia University Poison and Dependence Control Center in Egypt over two-year period. The study collected various types of data on each patient, such as demographic information, clinical examination results, laboratory investigations, ECG, and echocardiography. Logistic regression analysis and the SPSS package were used to identify predictors of mortality in patients with acute aluminum and zinc phosphide poisoning. The dataset used in the study consisted of 399 patients with acute aluminum and zinc phosphide poisoning, collected over two yearsch 124 patients did not survive. Demographic and poison data were collected for each patient, as well as clinical examination results, laboratory investigations, ECG, and echocardiography
^30^	Logistic regression analysis and SPSS package	This study aimed to determine clinical and laboratory markers that could help in predicting the medical outcome of acute AlP poisoning cases using established scoring systems in Iran. A total of 68 confirmed cases were evaluated, and 36 patients did not survive. Multiple logistic regression analysis revealed that four factors were significant predictors of mortality, namely Glasgow coma score (GCS), systolic blood pressure (SBP), urinary output (UOP), and serum (HCO_3_). A four-variable risk-prediction nomogram was developed to identify high-risk patients and predict the risk of mortality
^31^	Logistic regression analysis and SPSS package	The goal of the research was to assess the outcomes and prognostic indicators of acute aluminum phosphide (AlP) poisoning in Egypt over a two-year span. The data analyzed included 399 individuals with acute aluminum and zinc phosphide poisoning, with those who did not survive being more likely to have ingested aluminum phosphide tablets and having higher poisoning severity scores. The study involved a prospective analysis of 30 patients who were admitted to Alexandria Main University Hospital with acute AlP poisoning within a six-month duration. The data gathered included biographical data, medical history, poisoning history, medical examination, investigations, duration of hospitalization, and outcome
^32^	Logistic regression analysis and SPSS package	This study is a retrospective analysis of the medical records of 105 patients who were admitted to the Tanta Poison Control Unit with acute AlP poisoning from January 2012 to December 2016, covering a five-year period. The primary objective of the study was to identify factors that could predict mortality and evaluate their associations with patient outcomes.The dataset collected for the study included sociodemographic data, toxicological data, physical examination results, laboratory investigations, and therapeutic interventions
^33^	SPSS package	The main focus of this study was to examine the patterns of intoxication, assess the role of antifibrinolytics in management, and recommend prevention and education programs for acute zinc phosphide poisoning in Egyptian patients. The dataset used in the study comprised 188 cases of such poisoning that occurred over a period of 22 months and were reported at the National Egyptian Center of Clinical and Environmental Toxicological Research

## Basics and background

### Relevance vector machine

RVM is a probabilistic model used for classification and regression tasks. RVM has gained popularity in recent years due to its ability to provide accurate predictions with a sparse set of relevant features.RVM is a type of SVM that uses Bayesian inference to estimate the hyperparameters of the model, which allows for automatic selection of the relevant features and regularization of the model. RVM is a sparse Bayesian model that selects only the most relevant features from the input data, which helps to reduce overfitting and improve generalization. While SVM is a non-probabilistic model that tries to find a hyperplane that separates the data into different classes. So the RVM has some advantages over SVM, such as being more computationally efficient and providing probabilistic outputs.

The RVM is a type of sparse linear model in which the basic functions are constructed by a $\varnothing$ kernel function for the training set $X=\left\{{x}_{1}, {x}_{2}, \dots {x}_{N}\right\}$^34^:

1 $$y\left(x\right)=\sum_{i=1}^{N}{w}_{i}\mathrm{\varnothing }\left(x-{x}_{i}\right)$$

where $y$ a prediction is function and $w=\left\{{w}_{1}, {w}_{2}, \dots {w}_{N}\right\}$ is weight vector.

Multi-kernel RVM is an extension of the simple RVM model. It consists of numerous different types of kernels ${\varnothing }_{m}$, as indicated by:

2 $$\mathrm{y}\left(\mathrm{x}\right)= \sum_{\mathrm{m}=1}^{\mathrm{M}}\sum_{\mathrm{i}=1}^{\mathrm{N}}{\mathrm{w}}_{\mathrm{mi}}{\mathrm{\varnothing }}_{\mathrm{m}}\left(\mathrm{x}-{\mathrm{x}}_{\mathrm{i}}\right)$$

The type of kernel functions is not restricted in RVM modeling. Meanwhile, empirically selecting the proper kernel function and determining the relevant hyper-parameter values is required. Therefore, the proposed model uses the optimization phase to select the optimal kernel function with its optimal hyper-parameters from different kernel functions.

### Bayesian optimization

Bayesian optimization is a type of sequential optimization learning approach that is commonly used for uncertain objective functions and can be expressed as equation^35^,

3 $${x}^{*}=\underset{x\in X}{\mathrm{argmax}}\ f \left(x\right)\ where\ X\subseteq {R}^{D}$$

The aim is to find the parameters that maximize function f(x) within a domain X that has finite lower and upper bounds for each variable. In contrast to grid search or random search, BO is a sequential search method that combines exploration and exploitation and is typically more efficient. BO consists of two main elements: an acquisition function that selects the next sample point, and a Bayesian statistical model that models the objective function. The Bayesian posterior probability distribution, usually a Gaussian process, predicts potential values of f(x) at a candidate point x and is updated every time f is observed at a new location. The acquisition function balances two important factors: exploring areas where the function has high uncertainty and exploiting areas where the function has strong predictive means^36,37^. If the Gaussian process is used to optimize the hyperparameters function ${\varvec{f}}\left({\varvec{x}}\right)$ uses the Gaussian process, then is ${\varvec{p}}\left({\varvec{f}}\left({\varvec{x}}\right)|{\varvec{x}},{\varvec{H}}\right)$ a normal distribution. Based on the results of existing N group experiments, ${\varvec{H}}={\left\{{{\varvec{x}}}_{{\varvec{n}}},{{\varvec{y}}}_{{\varvec{n}}}\right\}}_{{\varvec{n}}=1}^{{\varvec{N}}}$, BO is modeled as a Gaussian process, and the posterior distribution ${\varvec{p}}\left({\varvec{f}}\left({\varvec{x}}\right)|{\varvec{x}},{\varvec{H}}\right)$ of ${\varvec{f}} \left({\varvec{x}}\right)$ is calculated^38^. The acquisition function, denoted as a(x,H), balances the selection of sampling points between regions with high predicted objective and regions with high prediction uncertainty. The function is utilized to determine the next sampling point by maximizing the acquisition function. In order to maximize the acquisition function, the next sampling point will be chosen accordingly.Assume that ${{\varvec{y}}}^{*}=\mathbf{m}\mathbf{i}\mathbf{n}{{\varvec{y}}}_{{\varvec{n}}}, 1\le {\varvec{n}}\le {\varvec{N}}$ is the optimal value in the currently existing sample, the desired improvement function is as follows^39^:

4 $$a\left(x,H\right)= \underset{-\infty }{\overset{\infty }{\int }}\mathrm{max}\left({y}^{*}-y,0\right)P\left(y|x,H\right) dy$$

Bayesian optimization employs a non-parametric Gaussian process as a probabilistic measure to model the objective function, resulting in robust optimization solutions. The acquisition function serves as a substitute utility function, directing the search for the next observation. It ensures a balance between exploring areas with high epistemic uncertainty about the function and exploiting areas with high predictive mean.

## Materials and methods

During the period from January 1st, 2017 to the end of December 2021, all cases of acute zinc and aluminum phosphide poisoning were considered for inclusion in the study. The diagnosis of phosphide intoxication was made based on the patient's history of exposure, which was obtained from the patient or accompanying relative, and the presence of symptoms and signs of phosphide intoxication upon admission. Patients with co-ingestion with other drugs, underlying chronic diseases such as diabetes mellitus and renal failure, and acute pathologies such as burns and trauma were excluded from the study.

The following information was collected from the patients' records: personal data (age, sex, residence, and occupation) and toxicological data (type of phosphide poison, route of administration, and mode of exposure). Additionally, a clinical evaluation was performed, including an assessment of initial vital signs (blood pressure and pulse rate), physical examination, and echocardiography. A silver nitrate paper test was conducted on the gastric content of all cases^40^, and serum cholinesterase levels were measured in all cases^41^. Thin-layer chromatography was performed when needed to exclude mixed toxicity with organophosphate or carbamate in cases with a positive silver nitrate test and decreased serum cholinesterase levels^42^.

### Ethical consideration

The Forensic Medicine and Clinical Toxicology Department and the Research Ethics Committee of Menoufia Faculty of Medicine approved the study, and the Declaration of Helsinki and National Guidelines were followed. To maintain confidentiality, the records were kept anonymous. The participants provided written informed consent to participate, and the committee confirms that all research was conducted ethically.

### Statistical method

Data analysis and presentation were carried out through SPSS version 22. To examine the correlation between two variables, Pearson's Chi-square test was employed, and the qualitative outcomes were represented as percentages and numbers in brackets. Linear regression analysis was utilized to forecast the value of one variable based on another variable. A significance level of p < 0.05 was established, and these findings were employed to interpret the test results^43^.

### Optimized predictive modeling

BO-RVM is an optimized predictive model that utilizes RVM for selecting relevant features and Bayesian optimization for tuning hyperparameters. It is especially beneficial in cases where there are numerous features, and it becomes challenging to manually select the most relevant ones. BO-RVM is capable of handling non-linear relationships between input and output variables, which can improve the model's accuracy. Furthermore, it can reduce overfitting by selecting only the relevant features, making it useful for dealing with noisy or irrelevant features.

The proposed model utilizes Bayesian optimization, a technique that utilizes probabilistic models to search for the optimal hyperparameters based on their performance on a validation set. This method is more efficient and effective than grid search or random search, as it avoids exhaustive evaluation of all possible hyperparameter combinations. By incorporating BO in the BO-RVM model, the optimal hyperparameters for the RVM model can be found, resulting in improved predictive accuracy. BO builds a probability model of the objective function and selects the optimal weight vector, kernel function, and hyperparameters of RVM to evaluate the prediction objective function. The proposed model aims to optimize the objective function using a Gaussian process as a probabilistic measure, with Bayesian Optimization (BO) offering robust solutions. The selection of the optimum kernel function involves considering several commonly used kernel functions such as Gaussian, Polynomial, Sigmoid, and Laplacian. The RVM kernel and hyperparameters are randomly chosen to initiate the training process of the classifier model, which is then evaluated in the subsequent phase. BO conducts a search for the global optimal solution over multiple iterations until the termination criteria are satisfied, determined by achieving the optimal fitness value or reaching the maximum number of iterations. The fitness function used to calculate the optimal fitness value is accuracy, with the aim of minimizing the classification error. The algorithm involved in the proposed method, outlining the steps, is provided in Algorithm 1.
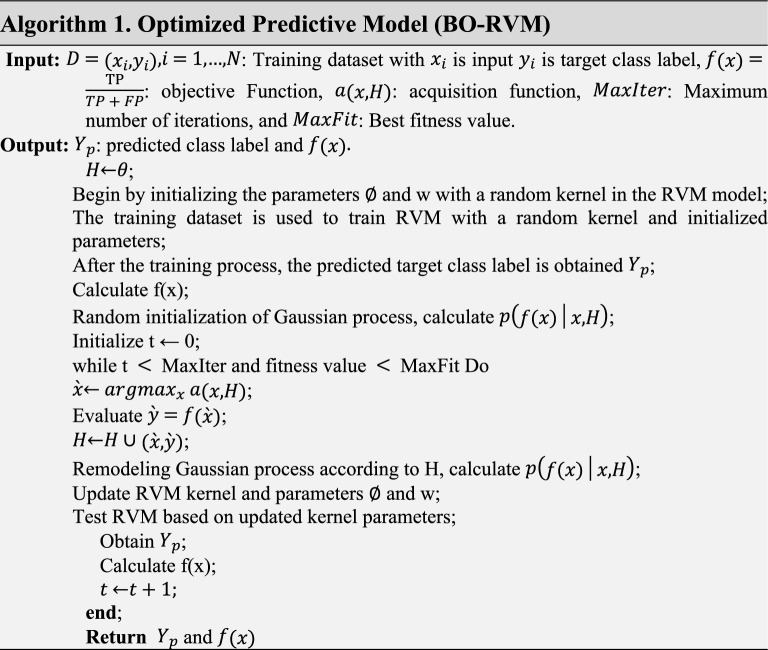


**Figure 1:**
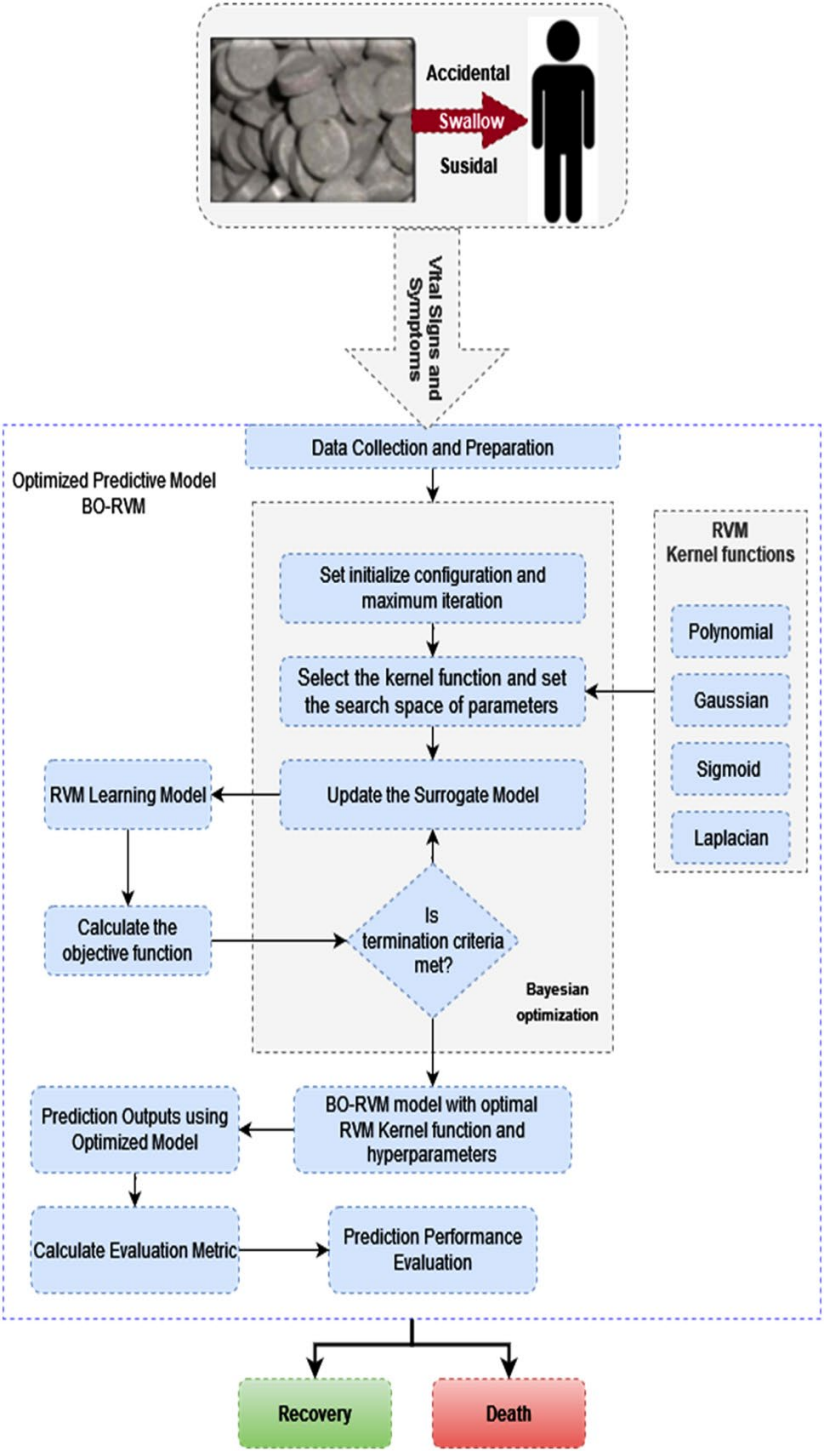
The proposed optimized predictive model (BO-RVM).

The process illustrated in Fig. 1 involves loading the dataset and initializing the RVM learning model with a random kernel and hyperparameters to commence the training process. The trained classifier model is then evaluated using a fitness function, which determines the classification error based on the prediction results from the confusion matrix. During each iteration, BO updates the RVM kernel function and hyperparameters until the optimal minimized classification errors are achieved, or the maximum number of iterations is reached. Ultimately, when the termination criteria are satisfied, the BO-RVM model is optimized with the optimal RVM kernel function and hyperparameters.

## Results, discussion, and analysis

### Statistical results and analysis

**Figure 2:**
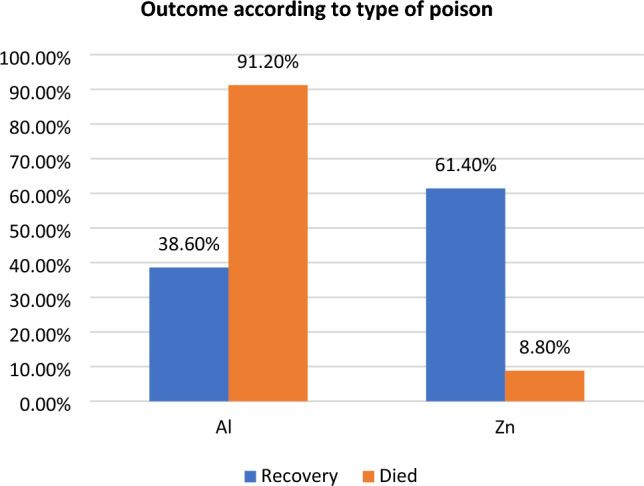
Distribution by poison type in studied patients.

The total number of all poisoned cases admitted to the Menoufia University Poison and Dependence Control center during the study period was 10,636. Aluminum and Zinc phosphide poisoning cases constituted 2518 cases, accounting for an average of 23.67%. The highest death rate was observed in 229 cases (33.6%) of aluminum and zinc phosphide poisoning in the year 2020, followed by 200 cases (29.3%) in 2021, while the lowest rate was 52 cases (7.6%) in 2017. Among the aluminum and zinc phosphide poisoning cases, 682 deaths were recorded, with 622 cases (91.2%) from AlP and 60 cases (8.8%) from ZnP. Figure 2 shows the percentage of patients with different outcomes (survived or died) in relation to the type of poison (AlP or ZnP).

The age of the poisoned patients ranged from 1 to 60 years, with a median age of 18 years (20.15 ± 13.699). The majority of patients were females (1502 or 59.7%), and more than 45% of cases (1193) were in the age group of 10–20 years. Most cases were from rural areas (2229 or 88.5%). More than 50% of cases were students (1324 or 52.6%). Table 2 shows that there was a statistically significant difference between the type of poison and age, sex, and outcome where P < 0.001.

**Table 2:** Distribution of the studied patients according to types of poison in relation to their characteristics (total number = 2518).

Items	Aluminum phosphide (no = 1330)		Zinc phosphide (no = 1188)		Χ^2^ and P-value
	No	%	No	%	
Age group					
< 10 years	252	18.9%	253	21.3%	51.430^HS^ P < 0.001
> 10–20 years	588	44.2%	605	50.9%	
> 20–30 years	156	11.7%	158	13.3%	
> 30–40 years	150	11.3%	95	8.0%	
> 40–50 years	87	6.5%	46	3.9%	
> 50 years	97	7.3%	31	2.6%	
Sex					
Female	742	55.8%	760	64.0%	17.46^HS^
Male	588	44.2%	428	36.0%	P < 0.001
Cholinesterase level					
Normal	395	29.7%	1159	97.6%	1222.98^HS^
Abnormal	935	70.3%	29	2.4%	P < 0.001
Silver nitrate test					
− ve	92	6.9%	1182	99.5%	2151.52^HS^
+ ve	1238	93.1%	6	0.5%	P < 0.001
Mode of poising					
Accidental	273	20.5%	266	22.4%	1.29^NS^
Suicidal	1057	79.5%	922	77.6%	0.255
Blood pressure					
Normal	708	53.2%	1128	94.9%	552.94^HS^
Abnormal	622	46.8%	60	5.1%	P < 0.001
Echo					
Normal	708	53.2%	1128	94.9%	552.94^HS^
Abnormal	622	46.8%	60	5.1%	P < 0.001
Pulse					
Normal	708	53.2%	1128	94.9%	552.94^HS^
Abnormal	622	46.8%	60	5.1%	P < 0.001
Outcome					
Recovery	708	53.2%	1128	94.9%	552.94^HS^
Died	622	46.8%	60	5.1%	0.000

Where NS: Means not significant, HS: Means highly statistical significance. P < 0.001: Means statistical significance, and No = number.

In 1979 cases (78.6%), poisoning was reported as having been caused by alleged suicidal intention. There was no statistically significant difference between the mode of poisoning and the type of poison. However, there was a highly significant relationship between the type of poison and pulse, blood pressure, echocardiography, cholinesterase level, and silver nitrate test with P < 0.001. Moreover, age and sex were also significantly related to the outcome with P < 0.001. The mode of poisoning was found to be significantly related to the outcome, and there was a highly significant relationship between pulse, blood pressure, echocardiography, cholinesterase level, silver nitrate test, and outcome with P < 0.001, as presented in Table 3.

**Table 3:** Distribution of the studied patients according to outcome in relation to their characteristics (total number = 2518).

Items	Survivors (no = 1836)		Died (no = 682)		Χ2 and P-value
	No	%	No	%	
Age group					
< 10 years	389	21.2%	116	17.0%	117.16^HS^ P < 0.001
> 10-20 years	904	49.2%	289	42.4%	
> 20–30 years	259	14.1%	55	8.1%	
> 30–40 years	156	8.5%	89	13.0%	
> 40–50 years	75	4.1%	58	8.5%	
> 50 years	53	2.9%	75	11.0%	
Sex					
Male	685	37.3%	331	48.5%	26.03^HS^
Female	1151	62.7%	351	51.5%	P < 0.001
Cholinesterase level					
Normal	1179	64.2%	375	55.0%	17.93^HS^
Abnormal	657	35.8%	307	45.0%	P < 0.001
Silver nitrate test					
− ve	1145	62.4%	129	18.9%	375.56^HS^
+ ve	691	37.6%	553	81.1%	P < 0.001
Mode of poising					
Accidental	414	22.5%	125	18.3%	5.26^S^
Suicidal	1422	77.5%	557	81.7%	0.022
Blood pressure					
Normal	1836	100.0%	0	0.0%	2518.00^HS^
Abnormal	0	0.0%	682	100.0%	P < 0.001
Echo					
Normal	1836	100.0%	0	0.0%	2518.000^HS^
Abnormal	0	0.0%	682	100.0%	P < 0.001
Pulse					
Normal	1836	100.0%	0	0.0%	2518.0^HS^
Abnormal	0	0.0%	682	100.0%	P < 0.001
Types of poison					
Aluminum phosphide	708	38.6%	622	91.2%	552.94^HS^
Zinc phosphide	1128	61.4%	60	8.8%	P < 0.001

Where HS: means highly statistical significance, P < 0.001: means statistical significance, and No = number.

Table 4 shows the Spearman correlation among outcome and types of poison and age, sex, and mode of poisoning. It revealed that there was a positive correlation between outcome and age, job, and mode of poisoning. Also, there was a positive correlation between the types of poison and sex. Meanwhile, there is a negative correlation between outcome and sex, type of poison and age. Table 5 shows the effect of the type of poison on cholinesterase level. It clarifies that cholinesterase level is dependent on the type of poison and was statistically significant (P < 0.001).

**Table 4:** Spearman correlation among outcome and types of poison and age, sex, job and mode of poising.

Items	Outcome		Types of poison	
	r	P-value	r	P-value
Age	0.177**	0.000	− 0.128**	0.000
Sex	− 0.102**	0.000	0.083**	0.000
Mode of poising	0.046*	0.022	− 0.023 ns	0.225

**Table 5:** Predicting effect of type of poison on cholinesterase level.

Model	Sum of squares	Df	Mean square	F	Sig
ANOVA^a^					
Regression residual total	761,487,054.868	1	761,487,054.868	1449.595	.000
	1,321,680,780.513	2516	525,310.326		
	2,083,167,835.381	2517			

Model	Unstandardized coefficients		Standardized coefficients	t	Sig
	B	Std. error	Beta		
Coefficients^a^					
(Constant) Types of poison	2611.749	44.967		58.081	.000
	− 1101.603	28.934	− 0.605	− 38.074	.000

^a^Dependent variable: cholinesterase.

^b^Predictors: (constant), types of Poison.

### Predictive results and analysis

Using MATLAB version 2022a with the machine learning and statistics toolbox, the BO-RVM model was proposed. The experiment involved a dataset of 2518 patients, with 1836 cases of recovery and 682 deaths. The data was prepared by scaling and normalization and was then divided into training and testing sets in an 80:20 ratio. The proposed predictive model determines the response of patients, either recovered or dead, based on six predictors including age, sex, type of poison, silver nitrate test result (positive or negative), cholinesterase level, and mode of poisoning (suicide or accidental).

The evaluation measures are presented in the form of a confusion matrix, which is commonly employed to showcase the performance of a classification model on a test set. This matrix maps predicted outputs to actual outputs, enabling the determination of correct predictions.The common evaluation measures are obtained from the confusion matrix to evaluate the predictive strength; Accuracy is the proportion of correct predictions among all predictions. PPV (positive predictive value) is the proportion of true positives among all positive predictions. Among all negative predictions, NPV (negative predictive value) represents the ratio of true negatives. TPR (true positive rate or sensitivity) is the proportion of true positives among all actual positives, while TNR (true negative rate or specificity) is the ratio of true negatives among all actual negatives. F1 score is a weighted mean of precision and recall, which balances the trade-off between them^44^.

**Figure 3:**
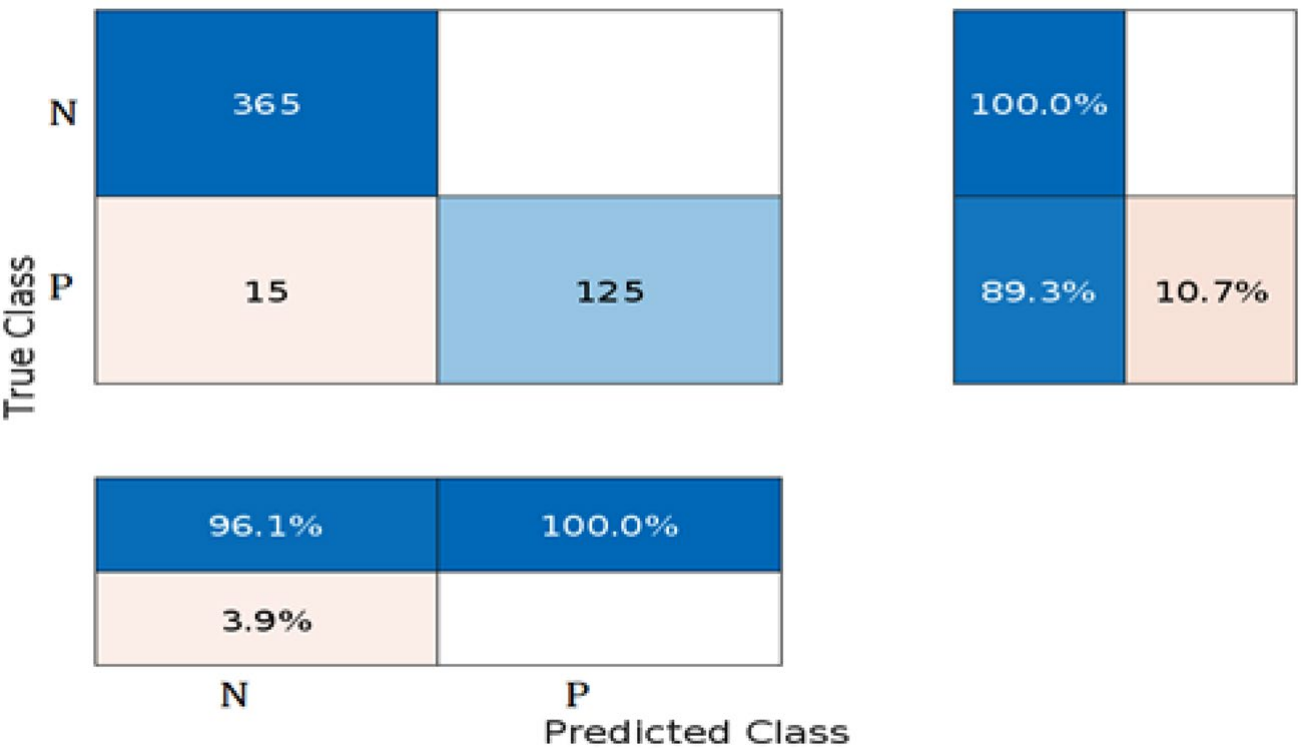
The confusion matrix of the proposed BO-RVM model.

**Figure 4:**
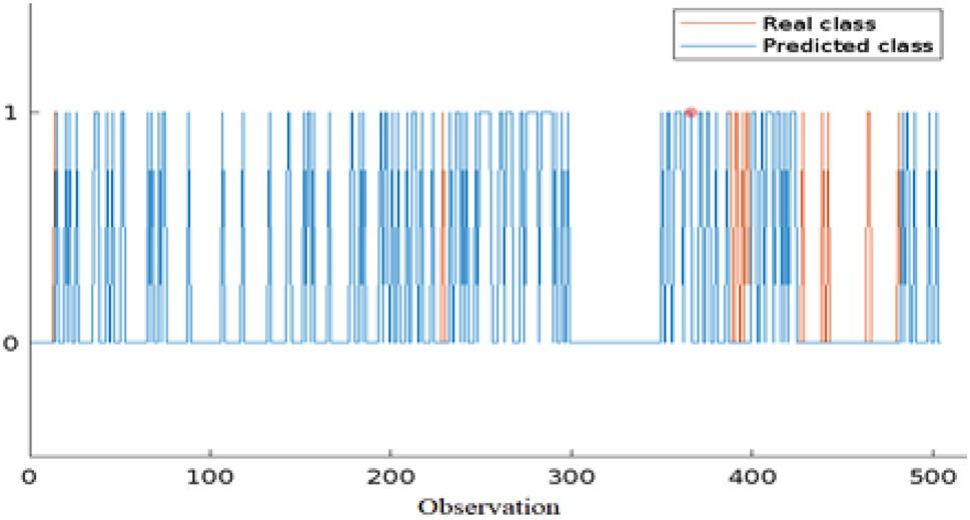
Observed vs. predicted values.

**Figure 5:**
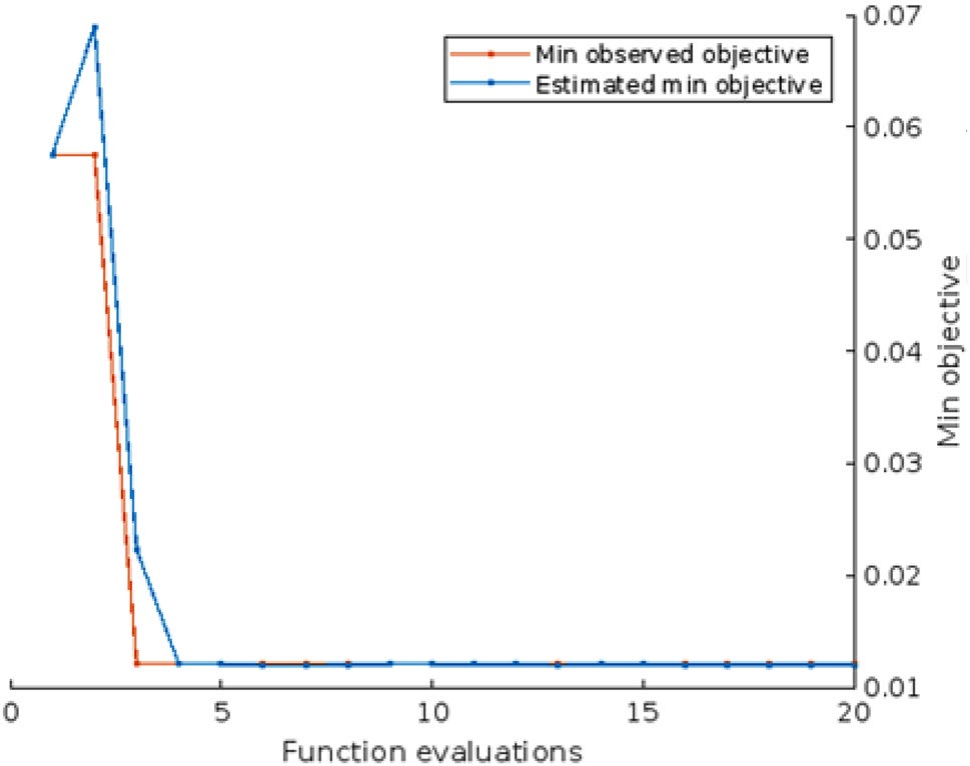
Min objective versus the number of function evaluation.

**Figure 6:**
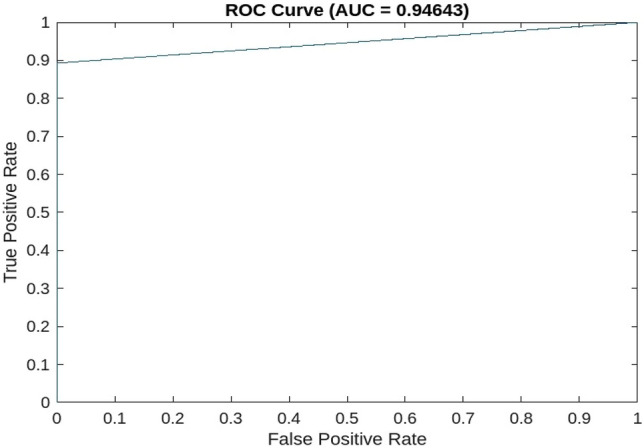
ROC AUC score of 0.94644.

The obtained results in the form of the confusion matrix are shown in Fig. 3. We assumed that "dead" is the positive class, while "recovered" is the negative class. The results indicate that the model has a high overall accuracy of 97%, which means that it correctly classified 97% of the cases. The model also has high PPV and NPV values of 100% and 96% respectively, indicating that it is good at predicting both positive and negative cases. The TPR or Sensitivity is also high at 89.3%, which means that the model correctly identified 89.3% of the positive cases. The TNR or Specificity is also high at 100%, indicating that the model correctly identified all negative cases. The F1 score of 94.3% indicates that the model has a good balance between precision and recall. Overall, these results suggest that the model is performing well in identifying both positive and negative cases. Figure 4 shows the compared results between the observed values and the corresponding predicted values. Additionally,  Fig. 5 the results of the optimization phase are represented by a plot of the minimal observed and estimated function values vs the count of function evaluations. The ROC AUC is presented in Fig. 6, score of 0.94644 indicates that the BO-RVM model has a high level of accuracy in predicting the target variable. This is a positive result and suggests that the model is effective in handling prediction problems. Additionally, the elapsed time of 379.9595 s for running the BO-RVM model is a valuable piece of information that highlights the efficiency of the proposed approach in terms of CPU time. This result suggests that the model can process data quickly and accurately, making it a promising tool for various applications.

### Discussion and limitations

Over a period of five years (2017–2021), 2518 patients with acute poisoning from zinc phosphide (ZnP) and aluminum phosphide (AlP) were admitted to Menoufia University Poison and Dependence Control Center. Among them, the death rate was 27.1%, which was similar to the results of studies conducted in India, Iran, and Albania^45–48^. The mortality rate from aluminum phosphide alone was 87.86% (622 out of 708 cases) and accounted for the majority of deaths among all phosphide-related deaths (622 out of 682 cases; 91.20%). In addition, acute toxicity from aluminum and zinc phosphide was the leading cause of death among all poisoning cases during the study period (682 out of 822 cases; 83% of total deaths). The results showed an uprising mortality rate from 17.7% in 2019 to 29.3% in 2021. The increasing rate of mortality is due to uncontrolled marketing, the cheapness of zinc and aluminum phosphide, lacking knowledge on social media about them. Moreover, during this period COVID-19 pandemics were present and lockdown help some cases to commit suicide using AlP. Many AlP-poisoned patients did not get proper supportive treatment. In fact, it is one of the most significant causes of fatal poisoning worldwide.

The findings of the study indicated that more than half of the cases were in the age group of 10–20 years, and the majority of the cases were females (59.7%). There was a notable distinction observed between the age and gender groups of the survivors and deceased cases. These groups tend to be more vulnerable to social and psychological stress, emotional problems, conflicts with others, and educational issues, which could potentially make them more prone to suicide^46,49^.

Moreover, most cases ingested phosphides in suicidal attempts, with a significant increase in the number of fatalities (81.7%). This is consistent with previous findings, such as a report by Konradsen et al.^50^ suicide accounted for 85% of fatal AlP cases. Suicidal behaviors can be influenced by various factors, including family conflicts, drug and alcohol addiction, social isolation, mental discomfort, physical conditions, financial problems, and workplace issues^47^. Phosphide compounds are easy to obtain and cheap, and have a quick onset of action and high fatality rates, which may lead individuals to use aluminum and zinc phosphide for suicide. Similar conclusions were reported by previous studies as in^51–53^.

The present study discovered that suicide by poisoning was a significant issue for females, possibly due to limited freedoms, harmful cultural practices, and frustration within the Egyptian community. Furthermore, the majority of cases (88.5%) were from rural areas, which is likely due to the agricultural nature of Menoufia governorate and the prevalence of grain storage in rural areas. These findings are consistent with previous studies, such as Kapoor et al.^45^, who reported that 65.1% of aluminum phosphide poisoning cases occurred in rural areas, and Parmar et al.^54^, who demonstrated that AlP is widely used in cultivating activities without restrictions, which could contribute to its incidence in rural regions. The study also revealed a significant association between the outcome and the type of phosphide poisoning, with most deaths occurring from AlP, which is more dangerous since it hydrolyzes to generate phosphine gas at a higher rate than ZnP. Additionally, there was a notable difference in age and gender between survivors and deceased cases, as these groups are more susceptible to social and psychological stress, emotional problems, conflicts with others, and educational issues, making them more vulnerable to suicide.

Previous studies^40,55^ have shown significant associations between survivors and death cases in terms of blood pressure, pulse, and echocardiography, which is consistent with our findings. These associations can be attributed to the massive loss of intravascular fluid caused by vascular wall insufficiency after phosphine gas absorption, leading to hypovolemic shock^5,56^, as well as the direct cardio-toxic effects of phosphine gas that can induce profound circulatory collapse^57^. Moreover, our study found a highly significant correlation between positive silver nitrate screening tests for aluminum phosphide and deaths compared to survivors, with 93.1% of cases testing positive. The silver nitrate test is a chemical qualitative color test commonly used in clinical and forensic tests to detect phosphine (PH_3_) in stomach contents. In fact, a previous study^58^ suggested that the silver nitrate test is sensitive enough to detect very low concentrations of PH_3_, as low as 0.05 mg L^−1^ (0.05 ppm). The high significance of positive silver nitrate screening tests in deaths may be attributed to dead cases ingesting more of the poison, leading to a higher concentration of PH_3_ in their stomach content. Regarding cholinesterase levels, our study also showed a highly significant relation between survivors and death cases, with decreased cholinesterase levels occurring in 45% of deaths. This finding is consistent with the study by Saidi and Shojaie^4^, which demonstrated that plasma cholinesterase levels were inhibited in rats receiving AlP. Mittra et al.^59^ also confirmed that both AlP and phosphine can cause cholinesterase inhibition, although this inhibition is unlikely to be clinically significant.

This study has some limitations that need to be considered. Firstly, the sample size was small, and the participants were not diverse enough, which could limit the generalizability of the findings to other populations. Moreover, the data collected may have been subject to biases, which could affect the accuracy of the results. The single-center nature of the data collection further limits the external validity of the study. To address these limitations, future studies should consider using more representative sampling methods and adjusting for potential confounding factors. Multi-center data or studies conducted in different populations could also help to validate the results. Lastly, to better understand prognostic factors, prospective studies with larger sample sizes are highly recommended.

## Conclusion and future work

In conclusion, this study suggests that mortality in patients with phosphide poisoning can be predicted based on the type of phosphide, aluminum, or zinc. The study identifies several mortality risk factors, including a young age group, rural regions, suicidal ingestion, low blood pressure, pulse, and echocardiogram. The proposed BO-RVM model provides reliable predictions for fatal poisoning using the collected dataset. These findings underscore the importance of implementing public health policies in Egypt that restrict the availability and use of phosphides and raise public awareness of their high mortality rate. Healthcare professionals should adopt more effective treatment methods for phosphide-poisoning patients. The public healthcare system should establish strict regulations for the sale of aluminum phosphide, particularly among the young age group more susceptible to suicide. Future studies with larger sample sizes are recommended to better assess prognostic factors. Preventing poisoning is always preferable to management, and manufacturers should be encouraged to package tablets in non-ingestible plastic containers with small holes. Finally, the use of aluminum phosphide should be regulated or supervised by the Ministry of Agriculture (Supplementary Information S1).

## Supplementary Information

Supplementary Information 1.

Supplementary Information 2.

## Data Availability

All data generated or analysed during this study are included in this published article [and its supplementary information files].
