# Supplementary material for: Pattern and predictors of death from aluminum and zinc phosphide poisoning using multi-kernel optimized relevance vector machine
Source: Sci Rep. 2023 May 22;13:8268. doi: 10.1038/s41598-023-34489-x (PMC10201496; doi:10.1038/s41598-023-34489-x)
Supplement: Supplementary file 2 — Supplementary Information 2. [file 41598_2023_34489_MOESM2_ESM.docx]

Table 1: Percentage of distribution and mortality rates of Aluminum and Zinc phosphides poisoned cases admitted from 2017 to 2021. (Total number = 2518)

| Year | Total of Aluminum and Zinc phosphides poisoned cases | | Total of survivors (no= 1836) | | Total mortality cases (no= 682) | | Aluminum phosphide poisoned cases (no= 1330) | | Zinc phosphide poisoned cases (no=1188) | |
| --- | --- | --- | --- | --- | --- | --- | --- | --- | --- | --- |
|  | No | % | No | % | No | % | No | % | No | % |
| 2017 | 314 | 12.5 | 262 | 14.3 | 52 | 7.6 | 97 | 7.3 | 217 | 18.3 |
| 2018 | 447 | 17.8 | 367 | 20.0 | 80 | 11.7 | 284 | 21.4 | 163 | 13.7 |
| 2019 | 625 | 24.8 | 504 | 27.5 | 121 | 17.7 | 332 | 25.0 | 293 | 24.7 |
| 2020 | 480 | 19.1 | 251 | 13.7 | 229 | 33.6 | 273 | 25.0 | 207 | 17.4 |
| 2021 | 652 | 25.9 | 452 | 24.6 | 200 | 29.3 | 344 | 25.9 | 308 | 25.9 |

no= number

Table 2. Distribution of the studied patients according to Types of poison in relation to their characteristics. (Total number = 2518).

| **Items** | | | **Frequency** | | **%** |  |
| --- | --- | --- | --- | --- | --- | --- |
| Age  X ± SD | | | 20.15 ±13.699 | | |  |
| **Age group** | | |  | |  |  |
| <10 y | | | 506 | | 20.09 |  |
| >10-20y | | | 1193 | | 47.4 |  |
| >20-30y | | | 314 | | 12.5 |  |
| >30-40 | | | 245 | | 9.7 |  |
| >40- 50 | | | 133 | | 5.3 |  |
| >50 years | | | 127 | | 5.0 |  |
| **Sex** | | |  | |  |  |
| Male | | | 1016 | | 40.3 |  |
| Female | | | 1502 | | 59.7 |  |
| **Job** | | |  | |  |  |
| No job | | | 479 | | 19.0 |  |
| Student | | | 1324 | | 52.6 |  |
| Housewife | | | 251 | | 10.0 |  |
| Farmer | | | 143 | | 5.7 |  |
| Driver | | | 37 | | 1.5 |  |
| Worker | | | 50 | | 2.0 |  |
| Solider | | | 81 | | 3.2 |  |
| Teacher | | | 78 | | 3.1 |  |
| Retired | | | 75 | | 3.0 |  |
| **Residence** | | |  | |  |  |
| Ruler | | | 2229 | | 88.5 |  |
| Urban | | | 289 | | 11.5 |  |
| **Outcome** | | |  | |  |  |
| Died | | | 682 | | 27.1 |  |
| Survivors | | | 1836 | | 72.9 |  |
|  | | |  | |  |  |
| Table 3. Distribution of the studied patients according to types of poison in relation to their characteristics. (Total number = 2518) | | | | | | |
| Items | Aluminum phosphide (no=1330) | | | Zinc phosphide (no=1188) | | Χ^2^ and P – value |
|  | No | % | | No | % |  |
| Age. Group | | | | | | |
| <10 y | 252 | 18.9% | | 253 | 21.3% | 51.430^HS^ |
| >10-20y | 588 | 44.2% | | 605 | 50.9% |  |
| >20-30y | 156 | 11.7% | | 158 | 13.3% | P<0.001 |
| >30-40 | 150 | 11.3% | | 95 | 8.0% |  |
| >40- 50 | 87 | 6.5% | | 46 | 3.9% |  |
| >50 years | 97 | 7.3% | | 31 | 2.6% |  |
| Sex | | | | | | |
| Female | 742 | 55.8% | | 760 | 64.0% | 17.46 ^HS^ |
| Male | 588 | 44.2% | | 428 | 36.0% | P<0.001 |
| Residence | | | | | | |
| Ruler | 1189 | 89.4% | | 1040 | 87.5% | 2.128 ^NS^ |
| Urban | 141 | 10.6% | | 148 | 12.5% | .145 |
| Outcome | | | | | | |
| Recovery | 708 | 53.2% | | 1128 | 94.9% | 552.94 ^HS^ |
| Died | 622 | 46.8% | | 60 | 5.1% | .000 |
| Cholinesterase level | | | | | | |
| Normal  Abnormal | 395  935 | 29.7%  70.3% | | 1159  29 | 97.6%  2.4% | 1222.98 ^HS^  P<0.001 |
| Silver nitrate test | | | | | | |
| - Ve | 92 | 6.9% | | 1182 | 99.5% | 2151.52^HS^ |
| + Ve | 1238 | 93.1% | | 6 | 0.5% | P<0.001 |
| Mode of poising | | | | | | |
| Accidental | 273 | 20.5% | | 266 | 22.4% | 1.29 ^NS^ |
| Suicidal | 1057 | 79.5% | | 922 | 77.6% | .255 |
| Job | | | | | | |
| No | 234 | 17.6 | | 245 | 20.6% | 55.44^HS^ |
| Student | 658 | 49.5% | | 666 | 56.1% |  |
| Housewife | 145 | 10.9% | | 106 | 8.9% | P<0.001 |
| Farmer | 82 | 6.2% | | 61 | 5.1% |  |
| Driver | 27 | 2.0% | | 10 | 0.8% |  |
| Worker | 40 | 3.0% | | 10 | 0.8% |  |
| Solider | 48 | 3.6% | | 33 | 2.8% |  |
| Teacher | 36 | 2.7% | | 42 | 3.5% |  |
| Retired | 60 | 4.5% | | 15 | 1.3% |  |
| Blood pressure | | | | | | |
| Normal | 708 | 53.2% | | 1128 | 94.9% | 552.94 ^HS^ |
| Abnormal | 622 | 46.8% | | 60 | 5.1% | P<0.001 |
| Echo | | | | | | |
| Normal | 708 | 53.2% | | 1128 | 94.9% | 552.94 ^HS^ |
| Abnormal | 622 | 46.8% | | 60 | 5.1% | P<0.001 |
| Pulse | | | | | | |
| Normal | 708 | 53.2% | | 1128 | 94.9% | 552.94 ^HS^ |
| Abnormal | 622 | 46.8% | | 60 | 5.1% | P<0.001 |

NS: Means not significant, HS: Means highly statistical significance. P<0.001: Means statistical significance. No= number

Table 4. Distribution of the studied patients according to outcome in relation to their characteristics (Total number = 2518)

| Items | Survivors (no=1836) | | Died (no=682) | | Χ2 and P – value |
| --- | --- | --- | --- | --- | --- |
|  | No | % | No | % |  |
| Age. Group | | | | | |
| <10 y | 389 | 21.2% | 116 | 17.0% | 117.16^HS^ |
| >10-20y | 904 | 49.2% | 289 | 42.4% |  |
| >20-30y | 259 | 14.1% | 55 | 8.1% | P<0.001 |
| >30-40 | 156 | 8.5% | 89 | 13.0% |  |
| >40- 50 | 75 | 4.1% | 58 | 8.5% |  |
| >50 years | 53 | 2.9% | 75 | 11.0% |  |
| Sex | | | | | |
| Male | 685 | 37.3% | 331 | 48.5% | 26.03 ^HS^ |
| Female | 1151 | 62.7% | 351 | 51.5% | P<0.001 |
| Residence | | | | | |
| Ruler | 1621 | 88.3% | 608 | 89.1% | .362 ^NS^ |
| Urban | 215 | 11.7% | 74 | 10.9% | .547 |
| Cholinesterase level | | | | | |
| Normal | 1179 | 64.2% | 375 | 55.0% | 17.93^HS^ |
| Abnormal | 657 | 35.8% | 307 | 45.0% | P<0.001 |
| Silver nitrate test | | | | | |
| - Ve | 1145 | 62.4% | 129 | 18.9% | 375.56^HS^ |
| + Ve | 691 | 37.6% | 553 | 81.1% | P<0.001 |
| Mode of poising | | | | | |
| Accidental | 414 | 22.5% | 125 | 18.3% | 5.26 ^S^ |
| Suicidal | 1422 | 77.5% | 557 | 81.7% | .022 |
| Blood pressure | | | | | |
| Normal | 1836 | 100.0% | 0 | 0.0% | 2518.00 ^HS^ |
| Abnormal | 0 | 0.0% | 682 | 100.0% | P<0.001 |
| Echo | | | | | |
| Normal | 1836 | 100.0% | 0 | 0.0% | 2518.000 ^HS^ |
| Abnormal | 0 | 0.0% | 682 | 100.0% | P<0.001 |
| Pulse | | | | | |
| Normal | 1836 | 100.0% | 0 | 0.0% | 2518.0^HS^ |
| Abnormal | 0 | 0.0% | 682 | 100.0% | P<0.001 |
| Types of poison | | | | | |
| Aluminum phosphide | 708 | 38.6% | 622 | 91.2% | 552.94^HS^ |
| Zinc phosphide | 1128 | 61.4% | 60 | 8.8% | P<0.001 |
| Job | | | | | |
| No | 360 | 19.6% | 119 | 17.4% |  |
| Student | 1013 | 55.2% | 311 | 45.6% | 118.75^HS^ |
| Housewife | 178 | 9.7% | 73 | 10.7% |  |
| Farmer | 96 | 5.2% | 47 | 6.9% |  |
| Driver | 15 | 0.8% | 22 | 3.2% | P<0.001 |
| Worker | 15 | 0.8% | 35 | 5.1% |  |
| Solider | 63 | 3.4% | 18 | 2.6% |  |
| Teacher | 64 | 3.5% | 14 | 2.1% |  |
| Retired | 32 | 1.7% | 43 | 6.3% |  |

HS: Means highly statistical significanc: P<0.001: Means statistical significance. No= number

Table 5: Spearman correlation among outcome and Types of poison and age, sex, job and mode of poising.

| Items | Outcome | | Types of poison | |
| --- | --- | --- | --- | --- |
|  | r | P. value | r | P. value |
| Age | .177** | .000 | -.128 ** | .000 |
| Sex | -.102 ** | .000 | .083 ** | .000 |
| Mode of poising | .046 * | .022 | -.023ns | .225 |

Table 6: Predicting effect of type of poison on Cholinesterase level

| ANOVA^a^ | | | | | |
| --- | --- | --- | --- | --- | --- |
| Model | Sum of Squares | Df | Mean Square | F | Sig. |
| Regression  Residual  Total | 761487054.868 | 1 | 761487054.868 | 1449.595 | .000 |
|  | 1321680780.513 | 2516 | 525310.326 |  |  |
|  | 2083167835.381 | 2517 |  |  |  |
| a. Dependent Variable: Cholinesterase | | | | | |
| b. Predictors: (Constant), types of Poison | | | | | |
| Coefficients^a^ | | | | | |
| Model | Unstandardized Coefficients | | Standardized Coefficients | t | Sig. |
|  | B | Std. Error | Beta |  |  |
| (Constant)  Types of poison | 2611.749 | 44.967 |  | 58.081 | .000 |
|  | -1101.603 | 28.934 | -.605 | -38.074 | .000 |
| a. Dependent Variable: Cholinesterase | | | | | |

|  | Dependent Variable: Colinstrase |
| --- | --- |
| Colinstrase | 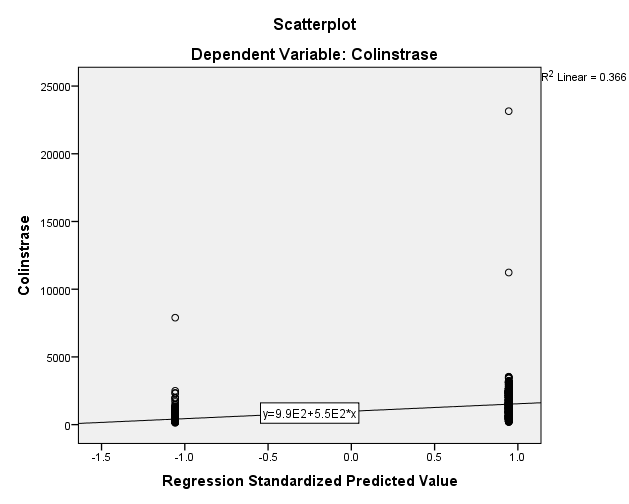 |
|  | Regression Standardized Predicted Value |

Fig 4. Predicting the effect of type of poison on Cholinesterase level
